# The Board Faultlines and Corporate Innovation Strategies Under the Influence of Property Rights Background and Institutional Environment

**DOI:** 10.3389/fpsyg.2022.857886

**Published:** 2022-04-25

**Authors:** Yan Zhang, Lianfu Ma

**Affiliations:** ^1^Research Center for Environment and Sustainable Development of the China Civil Aviation, Civil Aviation University of China, Tianjin, China; ^2^Business School, Nankai University, Tianjin, China

**Keywords:** social-related faultlines, cognitive-related faultlines, innovation strategy decision, property right background, institutional environment

## Abstract

This study takes the Chinese technology-intensive listed companies from 2009 to 2019 as the research sample to study the relationship between board faultlines and innovation strategy decisions of companies, and examines the impact of property rights background and institutional environment on the above relationship from the perspective of external governance environment of Chinese-listed companies. The results show that social-related faultlines of the board of directors have a negative influence on corporate innovation strategy decisions; cognitive-related faultlines have a positive effect on corporate innovation strategy decisions. At the same time, this research proves that the property rights background and institutional environment have a regulating role in the relationship between board faultlines and innovation strategy decisions, and can play an active role in the board faultlines.

## Introduction

In recent years, scholars in the field of corporate governance at home and abroad have been deepening relevant studies on the board of directors. A large number of scholars mainly focus on the board of directors and the relationship between the board of directors and the company value or the company performance (Olson et al., 2006; Hutzschenreuter and Horstkotte, 2013). Among them, researchers pay more attention to the composition, size, and characteristics of the board of directors, as well as the influence of the board of directors’ shareholding status and the dual chairman/CEO on the performance of the company and value creation (Sur et al., 2013; Vandebeek et al., 2016; Gupta et al., 2018). However, in the practices of many companies, such problems are found. Although the composition and characteristics of the board of directors of a company are similar and the internal and external environment of the company’s operation is similar, there may be significant differences in the decision-making of the board of directors of a company, which may lead to major differences in the future performance of the company (Veltrop et al., 2015; Georgakakis et al., 2017). This reality shows that the existing studies still cannot fully explain the impact of the composition, size, and characteristics of the board of directors on corporate performance and value creation.

In recent years, China has achieved the second-largest economy in the world with its rapid development. However, a series of social and environmental problems have accompanied its economic and social development. The emergence of such problems as low utilization rate of resources and environmental pollution urgently requires the Chinese enterprises to improve their production technology level, formulate strategic decisions for innovative development, and facilitate the transformation of China’s economic growth mode (Bruton et al., 2021). In the current situation, the Chinese government attaches great importance to the formulation and implementation of innovation development strategy, and also promotes Chinese enterprises, especially scientific and technological innovation enterprises, to continuously increase their work in technology and management innovation (Zhang et al., 2021).

As a special form of group decision-making, the board of directors needs to carry out in-depth communication and obtain sufficient information in the process of decision-making. The interaction among the members in the process of decision-making has a significant impact on the result of decision-making. Therefore, it is necessary to open the “black box” of the board of directors on the basis of traditional research, and change from the traditional research on the board of directors’ decision-making results to the research on the board of directors’ decision-making process and behavior. At the same time, it is necessary to study the internal mechanism of the board of directors and explore the decision-making process and the mechanism of the board of directors. Although a large number of studies have studied the influence of differences in the composition, size, and characteristics of the board of directors on decision-making, this interpretation is not enough to reflect the process of decision-making within the board of directors. As the group faultlines can be used as the basis to understand and study the diversity composition and efficiency of the group, it has a good application in revealing the dynamic behavior of the group members (Van Peteghem et al., 2018; Richard et al., 2019). Therefore, in the study of the decision-making process of the board of directors, we can use the research ideas and methods of the group faultlines for reference and introduce the concept of group faultlines into the board of directors.

This study will explore and analyze three key issues in the decision-making process of board innovation strategy based on board faultlines. First, how the communication, information, and resource acquisition among the members of the board of directors take place. Second, how the board faultlines affects the company’s innovation strategy decision. Third, whether the external environment of the company, such as the property rights background and regional system environment of the company, has an impact on the relationship between the board faultlines and the company’s decision-making.

## Basic Theory and Research Hypothesis

### Generation, Concept, and Connotation of Board Faultlines

The research on board faultlines stem from the dilemma of board diversity and board heterogeneity. The diversity and heterogeneity of the board of directors mainly refers to the diversity and difference of the board members in terms of gender, age, race, professional background, specialty, personality, and values. However, domestic and foreign scholars often reach inconsistent or even contradictory conclusions on the diversity and heterogeneity of the board of directors. For example, some scholars have found in their studies on board heterogeneity and corporate value creation that the differences of board members’ gender and race have a positive impact on corporate value creation (Carter et al., 2007; Miller and del Carmen Triana, 2009). However, some scholars hold a different view that there is no significant correlation between the differences of board members in terms of gender, race, age, and the value creation of the company (Van der Walt and Ingley, 2003; Rose, 2007). This is because these studies do not study the co-existence of multiple characteristics of team members, but only study the diversity and heterogeneity of the board of directors based on demographic characteristics (Lau and Murnighan, 1998; Georgakakis et al., 2017). However, the group faultlines can be used to study the group differentiation caused by the diverse characteristic combination of group members, which has become a new perspective of researching group diversity, and this concept has been valued in recent years (Richard et al., 2019).

The definition of the group faultlines has been given by pioneering studies. The group faultlines is a set of imaginary dividing lines dividing the group into several sub-teams based on one or more characteristics of the group members (Lau and Murnighan, 1998). Therefore, the board faultlines divides the board of directors into several sub-teams by the combined characteristic index. Moreover, the interior of each sub-team is relatively homogeneous and the sub-teams are heterogeneous to each other. Each sub-team has different behavioral characteristics. They interact with each other in the process of activities within the board of directors, resulting in communication, disagreement, alienation, or contradiction (Vandebeek et al., 2016; Van Peteghem et al., 2018).

### Board Faultlines and Corporate Innovation Strategy Decisions

Innovation strategy refers to the overall planning and action of companies to carry out various innovation activities, which usually involves the improvement and innovation of companies’ products or services (Carpenter and Westphal, 2001). As the core of the corporate governance mechanism, the board of directors plays an important role in the allocation of strategic resources, the provision of creative thinking, and the establishment of connections with the outside world (Johnson et al., 2011). Traditional upper echelon theory holds that company decision-makers are the key to the success of company’s strategic decision-making and implementation, and their demographic characteristics and heterogeneity are the important factors influencing the strategic decision-making. The core of this theory is that the characteristics of the decision-making subject reflect their cognition, and then affect their decision-making (Hambrick, 2007). In other words, the innovation strategy decision of a company is related to the composition characteristics of the decision-makers of the company and the potential relationship based on the characteristics of board members (Gupta et al., 2018).

According to the concept and connotation of board faultlines, the purpose of using board faultlines is to divide the board members with combined characteristic indices, and then to study the characteristics, behavior process, and the results of different sub-teams. Therefore, how to select the combination characteristics to form different types of board faultlines becomes the key to study. Some studies believe that group faultlines can be divided based on the work-related characteristics and physiological characteristics of group members, forming task-related faultlines and physiological characteristics faultlines (Hutzschenreuter and Horstkotte, 2013). At the same time, this study also shows that task-related faultlines and physiological characteristics faultlines have an impact on the expansion strategy decision of companies (Hutzschenreuter and Horstkotte, 2013). There are some studies that divided the board faultlines into structural dimensions and cognitive dimensions from the perspective of legal sources of board members and different cognitive characteristics (Li and Zhou, 2014). In addition, some studies have also classified the group faultlines, some of which divided the board faultlines into shallow faultlines and deep faultlines based on the demographic characteristics, capability, and personality of board members (Molleman, 2005). Some studies divided the group faultlines into social categories and task-related faultlines from intra-group conflicts. According to this study, social classification faultlines and task-related faultlines have different functional mechanisms within the group, and the two types of faultlines are related to relationship conflict and task conflict, respectively (Choi and Sy, 2010). Based on the study of faultlines at home and abroad, the existing studies usually divide the faultlines according to the combined characteristic indices. The mechanism of action of different types of faultlines and their behavioral results are different. Therefore, considering the combined characteristic indices selected by scholars at home and abroad, in the division of faultlines, this study investigated the influence of board faultlines on the innovation strategy of Chinese-listed companies under the Chinese scenario. Finally, this study determined that the board faultlines was divided into social-related faultlines and cognitive-related faultlines from the two dimensions of social classification and cognitive ability of the board members.

The social-related faultlines refers to the faultlines formed by the social characteristics of the board members. These social characteristics, such as age, gender, ethnicity, or race, can be directly perceived by social groups and change little (Crişan-Mitra et al., 2015). When making innovative strategy decisions, the board members need to communicate with each other about innovative ideas, real-time information, etc., while the existence of social-related faultlines will affect the innovative strategy decisions from two aspects. First, according to the relevant research of social psychology, the cognition, attitude, and emotion formed by group members toward other members are derived from explicit social characteristics. According to the theory of social classification and social identity, individuals make self-examination and self-evaluation by comparing themselves with other individuals. When individuals are found to have similar characteristics with other individuals, differences between “inside group” and the “outside group” will be formed. Individuals show strong identification with “inside group” members and exclude “outside group” members (Messick and Mackie, 1989; Veltrop et al., 2015). The social-related faultlines formed by the social classification between board members will affect the interaction among the members, lead to prejudice and discrimination between the sub-teams of the board, and hinder the process of innovation strategy decision-making (Duft and Durana, 2020; Grant, 2021; Nica and Stehel, 2021). Second, the similarity attraction paradigm also explains the formation of social-related faultlines from another perspective. According to this paradigm, similar individuals can form strong attraction and promote communication and interaction between individuals. Individual differences reduce this attraction, leading to less communication and interaction (Hutzschenreuter and Horstkotte, 2013). At the same time, the more similar characteristics are between individuals, the higher is the degree of communication within such “inside group,” and the more obvious are the faultlines between sub-teams. Thus, it can be seen that the social characteristic of the board of directors will divide the board of directors into sub-teams with different social characteristics. The greater is the difference between sub-teams, the deeper is the faultlines. Faultlines lead to lack of communication and interaction among sub-teams, and produces prejudice and discrimination, which is ultimately unfavorable for the board of directors to make innovation strategy decisions of the company. To sum up, this study proposes the following hypothesis:

H1: Social-related faultlines has a negative impact on the innovation strategy decisions of company.

The cognitive-related faultlines refers to the faultlines caused by differences in the knowledge and views of board members due to differences in professional skills, knowledge background, and functional background (Tuggle et al., 2010; Li and Zhou, 2014). The more diverse the board members are in terms of their professional, intellectual, and functional backgrounds, the more abundant the professional knowledge and perspective they bring. This has a positive effect on the company’s innovation strategy. When a company makes innovation strategy decisions, board members will face and deal with a large number of different types of information and data. The existence of cognitive-related faultlines will help the board members to understand and absorb different types of market information and make innovation strategy decisions. Based on the hypothesis of cognitive diversity, the existing studies believe that cognitive diversity can bring advantages to group process and output, including creativity, decision-making quality, and problem-solving ability (Williams and O’Reilly, 1998; Li and Zhou, 2014). The differences of board members in professional skills, knowledge background, and functional background will help the members to generate innovative ideas due to the collision of ideas, and avoid the phenomenon of “group thinking” in the group decision-making process. At the same time, the perspective of cognitive information processing can also explain the faultlines formed by board members based on different cognitive abilities, which enable the board members to have different understandings of the company’s innovation strategy and hold different views on how to make decisions (Fan and Du, 2015; Richard et al., 2019). The cognitive-related faultlines increases the value of information possessed by board members, facilitate the flow, exchange, and sharing of knowledge and information among board members, and facilitate the company to form high-quality innovation strategy decisions. To sum up, this study proposes the following hypothesis:

H2: Cognitive-related faultlines has a positive impact on the innovation strategy decisions of company.

### Activation of Faultlines by Property Rights Background and Institutional Environment

Lau and Murnighan (1998) first proposed the concept of “activation of faultlines.” According to the research, there are several potential faultlines within the group, which do not always play a role, but are “activated” in a specific situation (Lau and Murnighan, 1998). For example, when the board discusses the decision-making of retirement and old-age care, the faultlines formed by the aggregation of age characteristics of directors will be stimulated and play a role. Similarly, when a company is faced with major problems, such as the introduction and distribution of scarce resources, the faultlines formed by the aggregation of functional characteristics of directors will be stimulated and play a role. Based on the previous studies, later scholars formally defined the concept of faultlines activation, and clearly proposed the two groups of concepts of “potential faultlines” and “activation of faultlines” (Jehn and Bezrukova, 2010). Some scholars have taken it a step further; they believed that the differences of individual characteristics are subconsciously influenced by some specific environment or factors in the group, and thus the division of teams is formed within the group. This process from the generation of differentiation awareness to the division of the group is the activation process of the faultlines (Bezrukova et al., 2010). But only when group faultlines are activated, these potential faultlines will affect the group’s behavior or decision-making and have an impact on the organizational performance (Ionescu, 2021). Other studies have expounded that the potential faultlines are only an objective internal division line, which does not have an actual impact on the group. They put forward “activation efficiency of faultlines,” which mainly elaborated the difficulty degree of various influencing factors to activate the faultlines (Fan and Du, 2015).

#### Activation of Property Rights

The property rights system can reflect the background and environment of the company. The property rights system mainly refers to a kind of institutional arrangement of the company’s property rights formed through the combination of the property rights relationship and the property rights rules, which can effectively organize and protect the company’s property rights.

In the practice of Chinese enterprises, the property rights system of the company is usually determined according to the attributes of the investors. Different investors have different property rights backgrounds. Different property rights of a company may lead to differences in performance. Due to the different property attributes of Chinese enterprises, companies with different property attributes will be subjected to government intervention in different degrees (Liu et al., 2003). Later, other Chinese scholars pointed out in their research that the differences in the property rights of a company cause different impacts on the decision-making behavior of the company. It can be seen that the property right background has a significant influence on the decision-making behavior of the company and other related operations (Li et al., 2011).

In the research on corporate property rights in China, the measurement of property rights is mainly about the division of property rights, and most of the research is about the classification of enterprise property rights into two categories, that is, state-owned enterprises and non-state-owned enterprises. In China, state-owned enterprises are large enterprises that are invested by the state or local governments and have a certain degree of control. State-owned enterprises are of great significance to the economic and social development, and they have two main functions. First, state-owned enterprises should serve the growth and development of the national economy and ensure the basic needs of the livelihood of people. Second, state-owned enterprises also need to guarantee the appreciation of state assets. Therefore, it can be known that state-owned enterprises need to be responsible for the country and the people, and their development direction and business philosophy will be subjected to government intervention to varying degrees. In contrast with state-owned enterprises, non-state-owned enterprises have no national or government background and have a higher degree of freedom in development and operation. They only have to meet the laws of the state and the rules of business. However, the major shareholders and founders of non-state-owned enterprises have a strong influence on the company. Therefore, non-state-owned enterprises are more deeply affected by the intervention and influence of major shareholders, company founders, and other individuals (Li et al., 2008).

The property rights background brings different external environment for the operation of the company. State-owned enterprises are more supported by government policies and funds, while non-state-owned enterprises lack the economic foundation of state-owned enterprises (Cooper et al., 2014). The company’s investment in the technological innovation and change is long-term and risky. To ensure the company’s technological innovation and change can be promoted continuously and achieve certain results, the company needs a large number of continuous resources as a guarantee. However, compared to state-owned enterprises, non-state-owned enterprises in China lack stable and long-term financial resource or information support, and the information barrier of companies in innovation and change is higher than that of the state-owned enterprises. However, non-state-owned enterprises have stronger driving force for innovation and reform, and the executive order has relatively few constraints on them. Considering the different property rights backgrounds of enterprises, the state-owned enterprises need to undertake more social functions, and the board of directors will be subjected to more policy intervention from the government in the decision-making process. In the process, the policy will of the government will guide and objectively require board members to make decisions in line with the positioning of state-owned enterprises. In contrast, non-state-owned enterprises have no such constraints and restrictions. In the decision-making process, the board members have more freedom of thought and will.

Based on the above analysis, this study proposes the following hypothesis:

H3a: The property rights background has a moderating effect on the relationship between social-related faultlines and innovation strategy decision-making. Compared to state-owned enterprises, the social-related faultlines have a stronger negative influence on the innovation strategy decision-making of non-state-owned enterprises.

H3b: The property rights background has a moderating effect on the relationship between cognitive-related faultlines and innovation strategy decision-making. Compared to state-owned enterprises, cognitive-related faultlines have a stronger positive influence on the innovation strategy decision-making of non-state-owned enterprises.

#### Activation of Institutional Environment

The corporate innovation strategy decision is a kind of high uncertain strategy decision. Therefore, the company needs a good market environment and competition to improve the predictability of innovation strategy decision-making results (Ma et al., 2016). The better the regional institutional environment is, the lower is the uncertainty degree of enterprise technological innovation and R&D risk; the higher the enthusiasm of enterprise technological innovation and product research are, the higher is the enterprise capital investment (Liu and Li, 2012). However, China has a vast territory, and its market environment varies greatly in different regions. The higher the degree of regional marketization, the more mature the regional legal system, factor market, and financial market will be, which can provide a fairer and orderly environment for enterprises to make innovative decisions (Zhang et al., 2021). On the one hand, in a market with a better institutional environment, individuals with different characteristics are more likely to express their personalities and attitudes, and the sub-teams divided by social-related faultlines are more obvious, which are not conducive to the formation of corporate innovation strategy. On the other hand, a good institutional environment can stimulate individuals’ awareness of innovation and recognition of innovation strategy decision-making. Besides, the activation theory of faultlines believes that the faultlines are objectives, but they need to be stimulated by specific factors; otherwise, the fault zone will be in a dormant state. The company’s institutional environment can be regarded as a motivating factor. The cognitive-related faultlines formed by board members with different professional skills, knowledge background, and functional background in the institutional environment will be stimulated. The cognitive-related faultlines prompt board members to have more views and discussions on the issue of corporate innovation, which is conducive to the formation of corporate innovation strategy decisions.

Based on the above analysis, this study proposes the following hypothesis:

H4a: The institutional environment negatively regulates the relationship between social-related faultlines and innovation strategy decision-making. The better the institutional environment the enterprise is in, the stronger is the negative influence of the social-related faultlines on the company’s innovation strategy decision.

H4b: Institutional environment positively regulates the relationship between cognitive-related faultlines and innovation strategy decision-making. The better the institutional environment the enterprise is in, the stronger is the positive influence of the cognitive-related faultlines on the company’s innovation strategy decision.

## Research Design

### Sample Selection and Data Sources

Considering the Chinese situation of the study, this study takes the Chinese technology-intensive enterprises as samples, and the classification of such enterprises has been done based on the study by Lu and Dang (2014). At the same time, considering that China’s listed companies have been required by China securities regulatory commission to disclose information about the company’s R&D expenditure, data of A-share listed companies in electronics, machinery, equipment, instruments, medicine, biological pharmacy, other manufacturing industries, and information technology industry from 2009 to 2019 are selected in this study. The company data needed for the research were collected from the annual report of each company and from the Chinese stock market and accounting research database. This study also supplements and evidences research data from authoritative media, such as Sina Net, Phoenix Net, and the annual reports of listed companies with the same board members. To improve the rigor of the study, the sample data are processed as follows:

First, we excluded ST and *ST (special treatment due to financial problems) companies and companies whose main business changes no longer belong to the above industries. Second, we excluded the incomplete disclosure of R&D data, financial data, and governance data in the database. Third, we excluded companies whose listing time is later than the research window. Finally, this study obtained 3,322 samples from 302 companies from 2009 to 2019. To eliminate the influence of extreme values, the continuous variable was treated with winsorized values at the level of 1%.

### Variable Definition and Measure

#### Dependent Variable

Innovative strategy decisions (*ISD*s). Based on the existing research (David et al., 2001; Olson et al., 2006), this study selects the company’s innovation investment as the proxy variable of innovation strategy decisions. The innovation investment of the company is mainly decided by the board of directors, which reflects the decision of the board of directors on resource allocation of the innovation strategy and is the direct result of the decision of the company’s innovation strategy (Cunningham, 2021; Galbraith and Podhorska, 2021; Kovacova and Lăzăroiu, 2021). There are two main measures of innovation investment (Daellenbach et al., 1999). The first is the scale of R&D investment, expressed in the natural logarithm of the amount of R&D expenditure. The second is the intensity of R&D investment, which is measured by the proportion of R&D expenditure in operating income, the proportion of R&D expenditure in total assets, or the proportion of R&D expenditure in the enterprise market value. As operating income is vulnerable to management manipulation and sample data are unreliable, the proportion of R&D expenditure in the total assets of the company is adopted to measure the level of innovation strategy decision-making.

#### Independent Variables

Social-related faultline (*SRF*). According to the above analysis, social-related faultlines are measured by the age, gender, race, and other characteristics of board members. However, given that the racial differences in the samples selected by this study are relatively small, racial characteristics are not used as a measurement factor for the rupture of social-related faultlines.

Cognitive-related faultline (*CRF*). Members with different professional backgrounds and education degrees in diverse groups can generate knowledge collision and integration (Pelled, 1996; Cooper et al., 2014; Richard et al., 2019). Therefore, this study selected professional background and education degree to measure cognitive-related faultlines.

The classic measurement method is used to measure the social-related faultlines and cognitive-related faultlines (Lau and Murnighan, 1998). According to the method, group faultlines are measured using a bisection pattern, which divides the group into two sub-teams according to the criteria. The reason is that when the group size is small, the group can hardly be divided into three or more sub-teams (Thatcher et al., 2003). Therefore, the equation for *SRF* and *CRF* is as follows:


(1) $$F\,a\,u_{\text{g}}=\frac{\sum_{j=1}^{p}\sum_{k=1}^{2}n_{k}^{\text{g}}\,{\left({\bar{x}}_{j\,k}-{\bar{x}}_{j}\right)}^{2}}{\sum_{j=1}^{p}\sum_{k=1}^{2}\sum_{i=1}^{n_{k}^{\text{g}}}{\left(x_{i\,j\,k}-{\bar{x}}_{j}\right)}^{2}}$$

g=1,2,3,…,S

For a board of directors with *n* members, the classification of faultlines is 2*^n^*^–1^ - 1. In Equation (1), *n* stands for the number of members on the board; *p* stands for the total number of features examined; *g* stands for the classification; $n_{k}^{g}$ represents the number of members in sub-team *k*, which is classified by way of *g*; ${\bar{x}}_{j}$ represents the average value of all board members on characteristic *j*; ${\bar{x}}_{j\,k}$ represents the average value of members in sub-team *k* on characteristic *j*; *x*_*ijk*_ represents the value of member *i* on characteristic *j* in sub-team *k*; *Fau*_*g*_ is the degree of board faultlines under the *g* classification and is between 0 and 1. The larger the value, the stronger are the faultlines, and vice versa.

#### Regulating Variables

Property rights background (*Own*). Chinese enterprises have different property rights systems according to different investors and actual control. According to the actual control of the company, this study divides the research object into two types of enterprises, which are state-owned enterprises and non-state-owned enterprises (including private, collective, foreign capital, and others). For this variable, the samples are grouped according to the actual control of the company. If the sample companies are ultimately controlled by state-owned enterprises, *Own* = 0. If the sample companies are ultimately controlled by non-state-owned enterprises, *Own* = 1.

Institutional environment (*Institute*). This study refers to the relevant research of Chinese scholars and measures the institutional environment of each region through quantitative measurement of market indices in China (Fan et al., 2011). Considering that this study used the data of technology-intensive enterprises from 2009 to 2019 as samples, but the index compiled by Fan et al. (2011) was not continuously updated. Therefore, this study uses the practice of Li et al. (2012) to replace the undisclosed data with the current data. In this study, the marketization index of each region is processed by calculating the average value of marketization index of each region first, and then grouping each region according to the value of marketization index higher or lower than the average value. The value of marketization index of the region higher than the average value is 1, and the value of marketization index of the region lower than the average value is 0. This study deals with the marketization index of each region. First, the study calculated the average value of each region’s marketization index. Then, each region was grouped according to the value of marketization index higher or lower than the average value. If the value of marketization index is higher than the average value, *Institute* is equal to 1, and if the value of marketization index is lower than the average, *Institute* is equal to 0.

#### Control Variables

Earnings of the previous year (*ROA_*t*–1_*). The earning situation of the previous year will have an impact on the corporate strategy (Geng and Wang, 2021; Grant, 2021).

Company size (*Size*). The company’s size is directly proportional to the company’s resources. The larger the company is, the more abundant the resources are, which can provide more support and guarantee for the company’s innovation (Liu and Li, 2012; Bruton et al., 2021).

Board size (*Bsize*). The size of the board of directors to some extent reflects the diversification level of the background of members in the board of directors, which may have an impact on the company’s innovation decisions (Sur et al., 2013; Zhang et al., 2021). This study takes it as a control variable and measures it with the number of board members at the end of the year.

Company growth ability (*Growth*). According to existing studies, a company’s ability to grow will also affect its innovation strategy. Andriopoulos and Lewis (2009) believed that the innovation of a company is positively correlated with the future growth of the company. High-growth companies pay more attention to innovation and tend to make a greater investment in innovation (Andriopoulos and Lewis, 2009). Therefore, the growth ability is selected as the control variable and included in the research model. The growth ability of the company is measured by the growth rate of its main business revenue.

Ownership concentration (*Herf*). According to the existing studies (Li et al., 2008; Cooper et al., 2014), the main components of the company’s major decisions have an important impact on the company’s innovation decisions. In view of this, this study considers the shareholding ratio of the first major shareholder (*Herf1*) and the shareholding ratio of the second-largest shareholder (*Herf2-10*) of the company as the measurement index of ownership concentration, and takes these two variables as control variables into the research model. In this study, the Herfindahl index method is selected for calculation. The calculation method of this index is shown in Equation (2):


(2) $$H\,\left(n\right)=\sum_{i=1}^{n}\theta^{2}$$

In Equation (2), *H*(*n*) stands for the degree of ownership concentration of the former *n* major shareholders, *θ^2^* stands for the square of the shareholding ratio of shareholder *i*, and $\sum_{i=1}^{n}$ stands for the square sum of the former *n* shareholder’s shareholding ratio. When *H*(*n*) *=* 1, all of the company’s equity is concentrated in the hands of the top *n* major shareholders. The larger the Herfindahl index, the more the company’s equity is concentrated among the top *n* largest shareholders.

Asset liability ratio (*Leverage*). According to relevant studies (Georgakakis et al., 2017; Van Peteghem et al., 2018), the company’s debt situation will have an impact on the company’s operation. When a company has a high level of debt, its creditors may have an influence on the company and thus control or intervene in the company’s free decision-making. Similarly, when the corporate debt levels are low, corporate creditors are less likely to intervene or influence corporate decisions. Therefore, this study chooses the company’s asset liability ratio as the control variable, and the calculation method of asset liability ratio is shown in Equation (3):


(3) Asset⁢liability⁢ratio=Total⁢liabilities/total⁢assetso⁢f⁢t⁢h⁢e⁢c⁢o⁢m⁢p⁢a⁢n⁢y×100%

Length of establishment of the company (*Age*). This study believes that the company’s innovation-decision is related to the life cycle of the company, and the development stage of the company will have an impact on the company’s innovation strategy decision. Therefore, this study selects the length of the establishment of the company to measure (Geng and Wang, 2021).

Year (*Year*). Considering the influence of different years, the time variables were controlled and 11 dummy variables from 2009 to 2019 were set (Geng and Wang, 2021).

The dependent variables, independent variables, regulating variables, and control variables are shown in Table 1.

**TABLE 1:** Main variables.

Variable type	Abbreviation	Variable name	Variable description
**Dependent**	*ISD*	Innovation strategy decision	Proportion of R&D cost to total assets
**Independent**	*SRF*	Social-related faultline	Investigation of age and sex characteristics
	*CRF*	Cognitive-related faultline	Investigation of professional background and educational level
**Regulating**	*Own*	Property rights background	For state-owned enterprises, the value is 0, for non-state-owned enterprises, the value is 1
	*Institute*	Institutional environment	Using the marketability index
**Control**	*ROA_*t*–1_*	Earnings of the previous year	Company’s earnings in the previous year
	*Size*	Company size	Total company capital
	*Bsize*	Size of the board of directors	Members of the board at the end of the year
	*Growth*	Company growth ability	Growth rate of the company’s main business income
	*Herf1*	Proportion of largest shareholder	Using the Herfindahl index
	*Herf2–10*	Proportions of second to 10th largest shareholders	Using the Herfindahl index
	*Leverage*	Asset liability ratio	Ratio of total liabilities to total amount of company assets
	*Age*	Length of establishment of the company	Time of company’s establishment
	*Year*	Year	The annual change

### Model Setting

To test the action mechanism of social-related faultlines and cognitive-related faultlines on innovative strategic decision-making, as well as the activation mechanism of property rights background and institutional environment on board faultlines, the following research model is established to test the research hypothesis proposed in this study, as shown in Equation (4):


(4) I⁢S⁢D=α+β⁢Ii⁢n⁢d⁢e⁢p⁢e⁢n⁢d⁢e⁢n⁢t⁢V⁢a⁢r⁢i⁢a⁢b⁢l⁢e⁢s+γ⁢Σj⁢C⁢o⁢n⁢t⁢r⁢o⁢l⁢V⁢a⁢r⁢i⁢a⁢b⁢l⁢e⁢s+ε

Among them, *ISD* is the dependent variable, representing the company’s innovation strategy decisions; *Independent Variables* represents social-related and cognitive-related faultlines; Σ*Control Variables* represents the control variables. *β_*i*_* is the coefficient of the explanatory variable; *γ_*i*_* is the coefficient of the control variable; α is the intercept term; and ε is the residual term. *Own* and *Institute* are tested by grouping, so there is no interaction term.

## Data Analysis and Results Discussion

### Descriptive Statistics and Correlation Test

The descriptive statistical results of the main variables in this study show that the mean value of *ISD* is 0.0219 and the standard deviation is 0.0251. The mean value of *SRF* is 0.5349 and the standard deviation is 0.0588. The mean value of *CRF* is 0.2261 and the standard deviation is 0.0910. At the same time, by testing the correlation coefficient of the main variables, the test results show that the social-related faultlines and cognitive-related faultlines were related to the company’s innovation strategy decision. In addition, the correlation between independent variables is relatively low, all of which are no more than 0.4. It is preliminarily proved that there is no serious multiple collinearity among the variables in the research model, which can be further studied. The correlation coefficient matrix of the sample company’s main variables is shown in Table 2.

**TABLE 2:** Correlation coefficient of each variable.

Variable	1	2	3	4	5	6
(1) *ISD*	1					
(2) *SRF*	−0.0928**	1				
(3) *CRF*	0.0763***	−0.0720*	1			
(4) *Bstock*	0.0726**	−0.0152*	0.0215**	1		
(5) *ROA_*t*–1_*	−0.0266**	−0.0274*	0.0267***	0.1710	1	
(6) *Size*	0.0555***	0.0393***	−0.0423**	0.0372**	0.0053**	1
(7) *Bsize*	0.0161**	0.0140**	−0.0156**	−0.0283**	0.0480**	0.0613***
(8) *Growth*	−0.0731***	0.0652**	0.0068*	−0.3139*	−0.2976*	0.0474**
(9) *Herf1*	−0.0370**	−0.0822**	0.0917**	0.0975*	0.1559**	0.0753**
(10) *Herf2–10*	−0.0428*	−0.0920*	−0.0017*	0.1030*	0.1323*	0.1271**
(11) *Leverage*	0.0016***	−0.042**	0.0864**	−0.1045*	0.0569**	−0.0888*
(12) *Age*	0.1294***	0.1426**	0.1426**	0.0215**	0.0040	−0.0553***

**Variable**	**7**	**8**	**9**	**10**	**11**	**12**

(7) *Bsize*	1					
(8) *Growth*	0.0606***	1				
(9) *Herf1*	0.1287***	0.1197**	1			
(10) *Herf2–10*	0.0996**	0.1013*	0.2774**	1		
(11) *Leverage*	−0.1266**	0.0536**	0.1823***	0.2093**	1	
(12) *Age*	−0.0743***	0.0283**	−0.3139*	−0.2987***	0.1530**	1

*Significant at 10% level; **significant at 5% level; ***significant at 1% level.

### Regression Analysis

In this study, multiple linear regression analysis was used in Stata 14.0 software to analyze the relationship between social-related faultlines, cognitive-related faultlines, and enterprise innovation strategy decision-making, as well as the influence of property rights background and institutional environment on the above relations. Considering the possible heteroscedasticity of the sample data, this study uses the ordinary least square regression of robust standard deviation modified to test the hypotheses. The stratification regression results are shown in Table 3. Model 1 only conducts regression analysis on control variables and innovation strategy decisions. Based on model 1, models 2 and 3 were, respectively, added into *SRF* and *CRF* for regression tests. The variance inflation factor (*VIF*) of all variables in the model was lower than 2, and the mean value was 1.35, which was significantly lower than the critical multicollinearity threshold of 10.0 recommended by Neter et al. (1996). It was proved again that the variables selected in this study did not have multicollinearity. Model 2 shows that the social-related faultlines (*SRF*) (β_*1*_ = −0.3933, *p* < 0.01) is significantly negatively correlated with the innovation strategy decision (*ISD*). Model 3 shows a significant positive correlation between cognitive-related faultlines *CRF* (β_*2*_ = 0.2088, *p* < 0.01) and innovative strategic decision (*ISD*). Therefore, the H1 and H2 of this study were verified.

**TABLE 3:** Board faultlines and innovation strategy decisions.

Variable	Model 1	Model 2	Model 3
*SRF*		−0.3933***	
		(−3.62)	
*CRF*			0.2088***
			(2.14)
*ROA_*t*–1_*	0.0001***	0.0001***	0.0000***
	(1.27)	(1.46)	(1.52)
*Size*	−0.0144**	−0.0139**	−0.0162**
	(−2.24)	(−2.60)	(−2.28)
*Bsize*	0.0029***	0.0035***	0.0019***
	(0.41)	(0.28)	(0.36)
*Growth*	0.0127**	0.0124	0.0139
	(0.44)	(0.42)	(0.57)
*Herf1*	0.0001***	0.0000***	0.0001***
	(3.31)	(3.00)	(3.27)
*Herf2–10*	0.0001***	0.0002***	0.0000***
	(5.38)	(5.76)	(5.61)
*Leverage*	−0.0160***	−0.0158***	−0.0160***
	(−5.39)	(−5.40)	(−5.36)
*Age*	−0.0001	−0.0000	−0.0000
	(−0.20)	(−0.19)	(−0.19)
*Year*	Control	Control	Control
*R* ^2^	0.3323	0.3630	0.3638
*Adj-R^2^*	0.3124	0.3362	0.3348
*F-value*	84.69***	87.43***	86.49***
*N*	3322	3322	3322

**Significant at 5% level; ***significant at 1% level.

Models 4 and 5 were used to examine the influence of the property rights background on the relationship between the social-related faultlines and the company’s innovation strategy decision. When the regression samples were state-owned enterprises, *Own* = 0, and when the confidence level was 90%, the social-related faultlines (*SRF*) was significantly negatively correlated with the company’s innovation strategy decision (*ISD*) (β_*1*_ = −0.3792, *p* < 0.1). When the regression samples were non-state-owned enterprises, *Own* = 1, and in 95% confidence level, the social-related faultlines (*SRF*) of the board was significantly negatively correlated with the company’s innovation strategy decision (*ISD*) (β_*1*_ = −0.3990, *p* < 0.05). By comparing the regression results of Models 4 and 5, it can be found that the confidence level of regression test increased from 90–95% in the samples of non-state-owned enterprises. It can be seen that the company’s property rights background is the activation factor of the board faultlines, which can effectively affect the influence of the social-related faultlines on the company’s innovation strategy decision. It can be concluded from Models 4 and 5, the property right background plays a regulating role in the relationship between the social-related faultlines and the innovation strategy decision-making. Compared with state-owned enterprises, the social-related faultlines has a stronger negative influence on the innovation strategy decision of non-state-owned enterprises. Therefore, the hypothesis H3a in this study can be verified.

Models 6 and 7 were used to examine the influence of the property rights background on the relationship between the cognitive-related faultlines and the company’s innovation strategy decision. When the regression samples were state-owned enterprises, *Own* = 0, and when the confidence level was 95%, the cognitive-related faultlines (*CRF*) was significantly positively correlated with the company’s innovation strategy decision (*ISD*) (β_*1*_ = 0.1326, *p* < 0.05). When the regression samples were non-state-owned enterprises, *Own* = 1, and in 99% confidence level, *t* the cognitive-related faultlines (*CRF*) was significantly positively correlated with the company’s innovation strategy decision (*ISD*) (β_*1*_ = 0.2339, *p* < 0.01). By comparing the regression results of Models 6 and 7, it can be found that the confidence level of regression test increased from 95 to 99% in the samples of non-state-owned enterprises. This result shows that the property rights background factor of the company is an activation factor of the board faultlines, which can promote the board cognitive-related faultlines to play an activation role and play an active role in the company’s innovative strategic decision-making. The test of Models 6 and 7 can prove that the property rights background has a regulating effect on the relationship between the cognitive-related faultlines and the innovation strategy decision. Compared to state-owned enterprises, the cognitive-related faultlines of the board have a stronger positive influence on the innovation strategy decision of non-state-owned enterprises. Therefore, the hypothesis H3b in this study can be verified. The details are shown in Table 4.

**TABLE 4:** Impact of property right background on the relationship between board faultlines and innovation strategy decisions.

Variable	Model 4	Model 5	Model 6	Model 7
	(*Own* = 0)	(*Own* = 1)	(*Own* = 0)	(*Own* = 1)
*SRF*	−0.3792*	−0.3990**		
	(−0.59)	(−3.50)		
*CRF*			0.1326**	0.2339**
			(2.24)	(2.69)
*ROA*_*t–1*_	0.0009***	0.0010***	0.0010**	0.0002***
	(1.24)	(1.30)	(1.10)	(1.73)
*Size*	−0.0091**	−0.0158**	−0.0136**	−0.0188**
	(−2.36)	(−2.60)	(−1.69)	(−1.50)
*Bsize*	0.0019**	0.0044**	0.0120**	0.0036***
	(1.48)	(1.69)	(0.28)	(0.49)
*Growth*	0.0119*	0.0141	0.0119*	0.0140
	(0.50)	(0.62)	(0.45)	(0.68)
*Herf1*	0.0001***	0.0000***	0.0001***	0.0001***
	(1.90)	(3.66)	(2.29)	(3.44)
*Herf2-10*	0.0002***	0.0002**	0.0001**	0.0002***
	(5.67)	(5.86)	(5.06)	(5.88)
*Leverage*	−0.0130*	−0.0127**	−0.0145**	−0.0176
	(−5.39)	(−5.57)	(−5.43)	(−5.40)
*Age*	−0.0000	−0.0000	−0.0000	−0.0001
	(−0.25)	(−0.18)	(−0.11)	(−0.30)
*Year*	Control	Control	Control	Control
*R* ^2^	0.2853	0.3914	0.2750	0.3418
*Adj-R^2^*	0.3025	0.3484	0.2813	0.3495
*F-value*	29.79***	62.32***	30.41***	38.91***
*N*	1375	1947	1375	1947

*Significant at 10% level; **significant at 5% level; ***significant at 1% level.

Models 8 and 9 were used to examine the impact of institutional environment on the social-related faultlines and the company’s innovation strategy decision. When the level of institutional environment is poor, *Institute* = 0, and in 95% of the confidence level, the social-related faultlines (*SRF*) was significantly negatively correlated with the company’s innovation strategy decision (*ISD*) (β_*1*_ = −0.3852, *p* < 0.05). When the level of institutional environment is good, *Institute* = 1, and in 99% of the confidence level, the social-related faultlines (*SRF*) were significantly negatively correlated with the company’s innovation strategy decision (*ISD*) (β_*1*_ = −0.3996, *p* < 0.01). Compared to the regression results of the two groups of samples, the confidence level of the regression test was improved from 95 to 99% in areas with a better institutional environment. This result shows that the institutional environment factor is an activation factor of the board faultlines, which can activate the further deepening of the influence of the social-related faultlines. Models 8 and 9 show that the institutional environment negatively regulates the relationship between the social-related faultlines and the innovation strategy decision. The better the institutional environment the enterprise is in, the more negative impact the social-related faultlines will have on the company’s innovation strategy decision. Therefore, the hypothesis H6a in this study can be verified.

Models 10 and 11 were used to examine the impact of institutional environment on the relationship between the cognitive-related faultlines and the company’s innovation strategy decision. When the level of institutional environment is poor, *Institute* = 0, in 90% of the confidence level, the board cognitive-related faultlines (*CRF*) were significantly positively correlated with the company’s innovation strategy decision (*ISD*) (β_*1*_ = 0.2487, *p* < 0.1). When the level of institutional environment is good, *Institute* = 1, under 99% of the confidence level, the cognitive-related faultlines (*CRF*) are significantly negatively correlated with the company’s innovation strategy decision (*ISD*) (β_*1*_ = 0.2839, *p* < 0.01). Compared to the regression results of the two groups, the confidence level of the regression test was improved from 90 to 99% in areas with a better level of institutional environment. This result shows that the institutional environment factor is an activation factor of the board faultlines, which can activate the further deepening of the influence of the cognitive-related faultlines. Models 10 and 11 show that the institutional environment positively regulates the relationship between the cognitive-related faultlines and the innovation strategy decision. The better the institutional environment the enterprise is in, the more positive impact the cognitive-related faultlines will have on the company’s innovation strategy decision. Therefore, the hypothesis H6b in this study can be verified. The details are shown in Table 5.

**TABLE 5:** Impact of institutional environment on the relationship between board faultlines and innovation strategy decisions.

Variable	Model 8	Model 9	Model 10	Model 11
	(*Institute = 0)*	(*Institute* = 1)	(*Institute = 0)*	(*Institute* = 1)
*SRF*	−0.3850**	−0.3996***		
	(−0.72)	(−3.25)		
*CRF*			0.2487*	0.2839***
			(1.22)	(2.20)
*ROA*_*t–1*_	0.0010**	−0.0010***	−0.0010**	−0.0001***
	(−1.39)	(−1.22)	(−1.38)	(−1.20)
*Size*	−0.0315**	−0.0310***	−0.0313**	−0.0177**
	(−1.72)	(−1.52)	(−1.66)	(−2.01)
*Bsize*	0.0042**	0.0045**	0.0033**	0.0007***
	(2.04)	(2.23)	(2.24)	(0.89)
*Growth*	−0.0108*	−0.0140*	−0.0116*	−0.0148
	(−0.56)	(−0.77)	(−0.65)	(−0.80)
*Herf1*	0.0001**	0.0000***	0.0001**	0.0001***
	(1.30)	(2.37)	(1.46)	(3.34)
*Herf2-10*	0.0001*	0.0002***	0.0002**	0.0001***
	(1.86)	(3.77)	(1.03)	(3.06)
*Leverage*	−0.0130*	−0.0128**	−0.0130*	−0.0122**
	(−5.38)	(−5.32)	(−5.35)	(−5.20)
*Age*	−0.0000	−0.0000	−0.0000	−0.0000
	(−0.16)	(−0.09)	(−0.09)	(−0.02)
*Year*	Control	Control	Control	Control
*R* ^2^	0.2748	0.3318	0.3546	0.3448
*Adj-R^2^*	0.3140	0.3183	0.2972	0.3100
*F-value*	27.69***	57.32***	23.69***	57.65***
*N*	1375	1947	1375	1947

*Significant at 10% level; **significant at 5% level; ***significant at 1% level.

### Robustness Check

To ensure the robustness of the research results, this study carried out the robustness test. The robustness test is mainly carried out from two aspects: the measurement of variables and endogenous control.

With regard to the remeasurement of dependent variables, this study selected the degree of R&D investment of the company as the alternative variable of the company’s innovation strategy decision. To test the stability, two methods were selected to measure the dependent variable. One is to use the ratio of corporate R&D expenditure to corporate operating income as a measure. The second method is to use the ratio of the company’s R&D expenditure to the company’s market value as a measurement method. It is found that the regression results are consistent with the results obtained in this study. With regard to the remeasurement of independent variables, this study remeasures the independent variables in the study separately. For the social-related faultlines, this study uses gender and working terms of directors to replace gender and age to calculate the faultlines. For the cognitive-related faultlines, this study uses the educational level and professional experience to replace the educational level and professional background. In this study, the regression test of the board faultlines by the new measurement method is carried out, and the test results are consistent with the previous regression results.

In endogenous control, considering the possible endogeneity between independent variables and dependent variables, dealing with data in a lag stage can solve this problem. Therefore, this study deals with board faultline data in a lag phase. Regression analysis shows that the research results are not affected.

At the same time, to eliminate the influence of the missing variables that do not change over time, this study adopts the fixed effect model at the company level to perform regression on the variables mentioned above. The re-regression analysis shows that the directivity of several regression coefficients among board faultline, innovation strategic decision, property rights background, and institutional environment has not changed, and they are all significant at the level of 0.05, which can verify the regression conclusion mentioned above. It can be seen that there is no serious endogenous problem between independent variables, dependent variables, and moderating variables.

The new analysis of the regression model proves that the conclusion of this study has certain stability and reliability.

### Regression Results Discussion

Regression analysis results verify the hypotheses H1 and H2 of this study. The greater is the degree of social-related faultlines, the more serious is the prejudice and discrimination between different sub-teams formed by the board of directors, resulting in the lack of in-depth communication and interaction within the board of directors. This is not conducive to in-depth analysis and discussion of problems in the strategic decision-making process of the board of directors, and ultimately is not conducive to innovation strategy decision-making. The greater is the degree of cognitive-related faultlines, the greater is the difference between different subteams formed by the board of directors, which can avoid the phenomenon of “group thinking” in decision-making, which facilitates the exchange and sharing of knowledge and information among the directors, and promote the formation of innovation strategy decision-making.

According to the regression analysis results of Models 4 and 5, the negative impact of social-related faultlines on the innovation strategy decision-making of non-state-owned enterprises is stronger than that of state-owned enterprises, and the hypothesis H5a proposed in this study is proved. The results confirmed that although the state-owned enterprises have a solid capital base and guarantee, can provide strong support for the company to choose innovation decision-making, but state-owned enterprises get more administrative constraints, need to take the necessary social services for economic and social development, such as functions, these factors affect the company’s innovation strategy. According to the regression analysis results of Models 6 and 7, cognitive-related faultlines has a stronger positive influence on the innovation strategy decision of non-state-owned enterprises than the state-owned enterprises, and the hypothesis H5b is proposed in this study is proved. The results of this study prove that in the non-state-owned property rights environment, the decision-making of the board of directors is less subjected to policy intervention, and the members of the board have more freedom of thinking and opinion. Therefore, the non-state-owned property rights background is conducive to the formation of the company’s innovative ideas.

According to the regression analysis of Models 8 and 9, the institutional environment negatively regulates the relationship between the social-related faultlines and the innovation strategy decision. This result supports Hypothesis H6a. The results show that in markets with better institutional environments, individuals with different characteristics are more likely to express their personalities and attitudes. The differences among sub-teams formed by social-related faultlines based on social characteristics are more obvious, which are not conducive to the formation of innovation strategy. This study proves that the institutional environment is the activation factor of the social-related faultlines. The stronger the institutional environment the company is in, the stronger is the negative impact of the social-related faultlines on the company’s innovation strategy decision. It can be seen from the regression analysis of Models 10 and 11 that the institutional environment positively regulates the relationship between the cognitive-related faultlines and the innovation strategy decision. This result supports the hypothesis H6b proposed in this study. The research results reflect that in an open and free-market environment, board members’ knowledge, skills, etc., will be activated, generating more new ideas and opinions, and the cognitive-related faultlines will be further activated. The stronger the institutional environment the company is in, the greater is the positive impact of the cognitive-related faultlines on the company’s innovation strategy decision.

## Conclusion and Enlightenment

This study verifies the relationship between the board faultlines and the company’s innovation strategy decision, which is of great theoretical and practical significance for the current lack of innovation-decision and lack of R&D investment in China’s technology-intensive enterprises. First, the board faultlines become an important variable to measure the board governance level after the traditional board composition and diversity study. At the same time, this study changes from the traditional research on the decision-making results of the board of directors to the research on the decision-making process of the board of directors and discusses the influence of bias, communication, interaction, information acquisition, and other behaviors in the decision-making process of the board of directors. Second, this study, respectively, discusses the influence of social-related faultlines and cognitive-related faultlines on innovation strategy decision-making. The research conclusion is helpful for technology-intensive enterprises to pay more attention to the governance of the board of directors, promote enterprises to form a reasonable level of social-related faultlines and cognitive-related faultlines, and constantly optimize the quality of director recruitment. Third, this study believes that the property rights of the company will play an active role in the influence of social-related faultlines and cognitive-related faultlines on the company’s innovation strategy decision. Compared to state-owned enterprises, the social-related faultlines has a stronger negative influence on the innovation strategy decision of non-state-owned enterprises, and the cognitive-related faultlines have a stronger positive influence on the innovation strategy decision of non-state-owned enterprises. In corporate practice, the board of directors should find an appropriate balance between negative and positive influences to ensure the level of innovation strategic decision-making. In terms of the innovation environment, it is found that the innovation environment is also an important factor influencing the decision-making of the board faultlines on the company’s innovation strategy. Therefore, governments at all levels and market regulatory departments should promote the degree of regional marketization, establish an orderly market pattern, create a fair and just competitive environment, and improve the level of innovation and marketization.

The research still has the following limitations. First, this study chooses to use the proportion of R&D expenditure in the total assets of the company to measure the company’s innovation strategy decision. This variable can also be considered from the number and proportion of the company’s R&D personnel and the number of patents applied by the company. In future research, these factors can be included into the measurement of innovation strategy decision-making. Second, through literature review and relevant theoretical analysis, this study selected two characteristic indicators of age and gender, educational level, and professional background of directors as the basis for the division of board faultlines. In fact, characteristics, such as directors’ values, personalities, and emotions, can be used to measure the board faultlines. However, considering factors, such as data acquisition, other possible measures are not adopted in this study. In fact, characteristics, such as directors’ values, personalities, and emotions, can be used to measure the board fault. However, considering factors, such as data acquisition, other possible measures are not adopted in this study. The inadequacies and limitations of the above studies will be the focus of future research, which needs to be further expanded in future research.

## Data Availability Statement

The raw data supporting the conclusions of this article will be made available by the authors, without undue reservation.

## Author Contributions

YZ proposed the research questions, designed the research scheme, collected data, conducted the statistical analysis, and wrote the draft of the manuscript. LM provided valuable suggestions and revised the manuscript. Both authors have approved the version of this manuscript.

## Conflict of Interest

The authors declare that the research was conducted in the absence of any commercial or financial relationships that could be construed as a potential conflict of interest.

## Publisher’s Note

All claims expressed in this article are solely those of the authors and do not necessarily represent those of their affiliated organizations, or those of the publisher, the editors and the reviewers. Any product that may be evaluated in this article, or claim that may be made by its manufacturer, is not guaranteed or endorsed by the publisher.
